# Whole-genome sequence of *Bacillus pseudomycoides* strain I32, a nitrous oxide-producing bacterium isolated from a woodchip bioreactor

**DOI:** 10.1128/MRA.00809-23

**Published:** 2023-11-20

**Authors:** Jeonghwan Jang, Satoshi Ishii

**Affiliations:** 1 Division of Biotechnology and Advanced Institute of Environment and Bioscience, Jeonbuk National University, Iksan, Jeonbuk, South Korea; 2 BioTechnology Institute, University of Minnesota, Saint Paul, Minnesota, USA; 3 Department of Soil, Water, and Climate, University of Minnesota, Saint Paul, Minnesota, USA; University of Southern California, Los Angeles, California, USA

**Keywords:** denitrification, woodchip bioreactor, *Bacillus*, nitrous oxide production, nitrate removal

## Abstract

We report here the draft whole-genome sequence of *Bacillus pseudomycoides* strain I32, a bacterium isolated from the denitrifying woodchip bioreactor and showing rhizoidal colony morphology with filamentous swirling pattern on the agar medium plate. The isolate produced nitrous oxide without known nitric oxide reductase genes on the genome.

## ANNOUNCEMENT

One of the denitrifying bioreactor bed types known as woodchip bioreactor is a subsurface trench filled with woodchips providing carbon and energy sources to denitrifying microbes (1, 2). To improve the nitrate-removing performance of woodchip bioreactors under low-temperature conditions, several nitrate-removing bacteria have been isolated (3, 4) from the field-scale bioreactors located near Willmar, Minnesota. A single colony of each of the isolates including *B. pseudomycoides* I32 was inoculated into the anaerobic culture tubes containing 5 mL R2A medium (BD Difco) supplemented with 5 mM nitrate and 10 mM acetate with N_2_:C_2_H_2_ (90:10) atmosphere in the headspace. After incubation for 5 days at 15°C, the concentrations of nitrate in the supernatant and nitrous oxide in the headspace were measured as described previously (4). The strain I32 produced the largest amount of nitrous oxide among the strains tested with no remaining nitrate in the culture medium (Table 1) and, therefore, considered as a candidate for bioaugmentation (5).

**TABLE 1:** N_2_O concentrations in the headspace of the anaerobic tubes cultured with the strains including *B. pseudomycoides* I32^*a*^

Strain	N_2_O concentration (ppm)
*Bacillus* sp. G20	335.80 ± 62.12
*Bacillus* sp. I18	295.22 ± 52.67
*Bacillus* sp. I29	184.22 ± 23.34
*B. pseudomycoides* I32	877.72 ± 72.84

^*a*^ Mean ± SD (*n* = 3) is shown.

Strain I32 was anaerobically grown in R2A medium overnight at 30°C, and the genomic DNA was extracted from the cell pellet by using the PowerSoil DNA Isolation Kit (MoBio Laboratories). A dual-indexed Nextera XT DNA library was created and used for the Illumina MiSeq 300 bp paired-end sequencing with V3 chemistry at the University of Minnesota Genomics Center (Minneapolis, MN). A total of 4,517,846 pass-filter (PF) reads (301 bp length) were obtained according to the manufacturer’s manual (6). Default parameters were used for all software described in this study unless otherwise specified. After low-quality PF reads and sequences on PF reads were filtered and trimmed, respectively, by using Trimmomatic ver 0.36 (7), *de novo* assembly was done by using Velvet ver. 12.0.8 (8) with a k-mer size of 121 bp, resulting in 499 contigs with sizes ranging from 1,005 to 239,073 bp and an N_50_ value of 23,679 bp. The assembled draft genome had a total length of 5,728,253 bp, and genome coverage was estimated to be 237-fold. Gene prediction and annotation were done using the NCBI Prokaryotic Genome Annotation Pipeline ver. 6.3 (9). Average nucleotide identity (ANI) values between strain I32 and other *Bacillus* sp. genomes were calculated using OrthoANIu (10).

**Fig 1:**
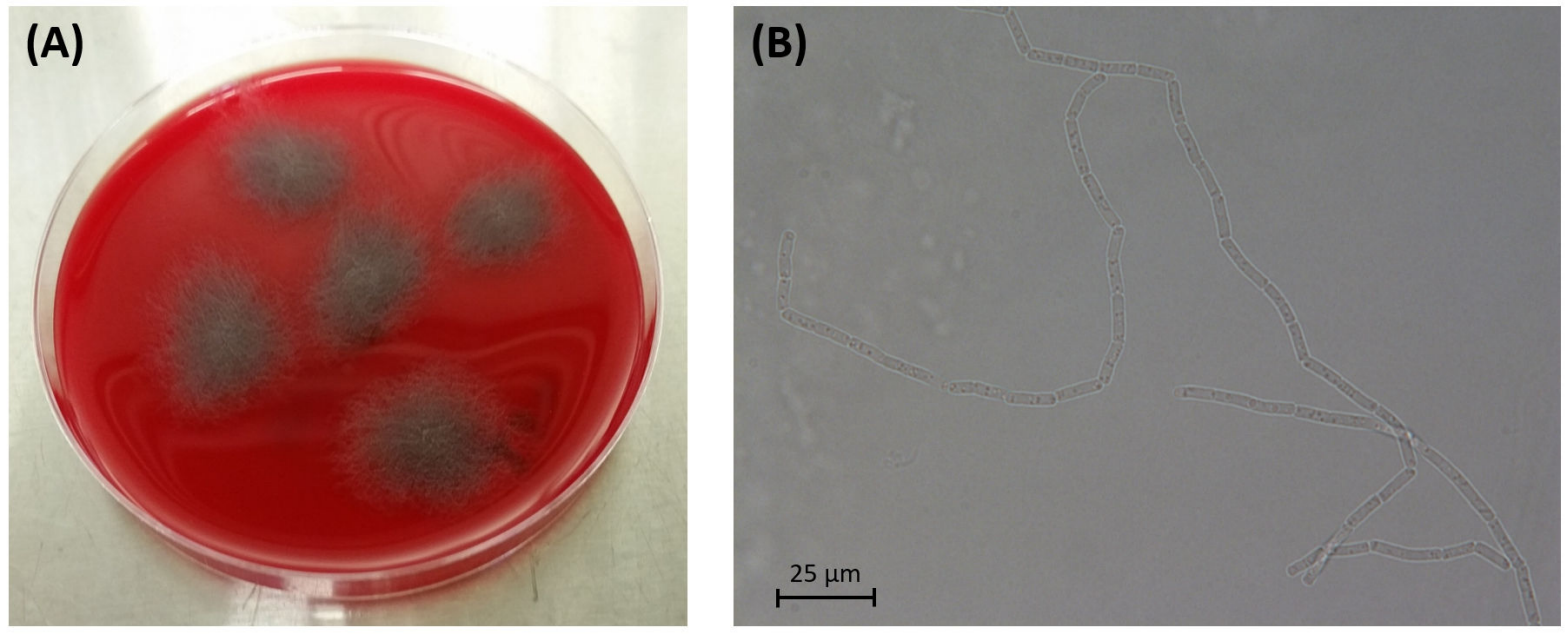
(**A**) Growth of *B. pseudomycoides* I32 showing rhizoidal colony morphology with filamentous swirling pattern on the Blood Agar plate [Triptic Soy Agar (Difco, BD) supplemented with 5% sheep blood]. (**B**) A microscopic image of strain I32 showing filamentous cell growth captured by a light microscope (Nikon 90i, Nikon Co., Ltd.).

The genome of strain I32 had a G + C content of 35.50% and contained 5,494 protein-coding sequences, 361 pseudogenes, 49 tRNAs, 3 partial rRNAs (two 5S and one 16S, incomplete assembly), and 5 noncoding RNAs. The ANI value between the genomes of strain I32 and the type strain of *Bacillus pseudomycoides* (DSM 12442^T^, GenBank accession number CM000745) was 98.59% with more than 60% genome coverages for both, which is greater than the species cutoff value (11). In addition, strain I32 was successfully detected by the *B. pseudomycoides*-specific TaqMan qPCR assay (12). It also showed the filamentous swirling colony morphology on agar plates (Fig. 1) similar to a previous study of *B. pseudomycoides* (13). All of these results suggest that strain I32 belongs to *B. pseudomycoides*. The whole genome sequence of strain I32 should help future studies, for example, to understand the metabolic pathways of nitrous oxide production without known nitric oxide reductase genes.

## Data Availability

The draft whole-genome sequence of *Bacillus pseudomycoides* strain I32 has been deposited in GenBank under the accession number MJAC00000000, and raw read sequence data are available with SRA accession number SRX22084641.
